# Targeting Immune Cells in the Tumor Microenvironment of HCC: New Opportunities and Challenges

**DOI:** 10.3389/fcell.2021.775462

**Published:** 2021-11-12

**Authors:** Xiaopei Hao, Guangshun Sun, Yao Zhang, Xiangyi Kong, Dawei Rong, Jinhua Song, Weiwei Tang, Xuehao Wang

**Affiliations:** ^1^ Hepatobiliary Center, The First Affiliated Hospital of Nanjing Medical University, Key Laboratory of Liver Transplantation, Chinese Academy of Medical Sciences, NHC Key Laboratory of Living Donor Liver Transplantation, Nanjing Medical University, Nanjing, China; ^2^ Department of General Surgery, Nanjing First Hospital, Nanjing Medical University, Nanjing, China

**Keywords:** HCC, TAM, NK cell, cancer immunotherapy, challenge, development

## Abstract

Immune associated cells in the microenvironment have a significant impact on the development and progression of hepatocellular carcinoma (HCC) and have received more and more attention. Different types of immune-associated cells play different roles, including promoting/inhibiting HCC and several different types that are controversial. It is well known that immune escape of HCC has become a difficult problem in tumor therapy. Therefore, in recent years, a large number of studies have focused on the immune microenvironment of HCC, explored many mechanisms worth identifying tumor immunosuppression, and developed a variety of immunotherapy methods as targets, laying the foundation for the final victory in the fight against HCC. This paper reviews recent studies on the immune microenvironment of HCC that are more reliable and important, and provides a more comprehensive view of the investigation of the immune microenvironment of HCC and the development of more immunotherapeutic approaches based on the relevant summaries of different immune cells.

## Introduction

Hepatocellular carcinoma (HCC) is the most common type of liver cancer, accounting for the sixth most common type of cancer and the second leading cause of death among all cancers (Ferlay et al., 2015). Due to changes in environmental factors, immunization and people’s lifestyle, the incidence of HCC and HCC-related mortality are increasing all over the world. The latest research indicates that HCC accounts for approximately 85% of patients diagnosed with liver cirrhosis. Its 5-year survival rate is only 18%, second only to pancreatic cancer (Asafo-Agyei and Samant, 2021). With the improvement of the treatment level for HCC, a variety of treatment options such as liver transplantation, surgical removal, systemic therapy and liver targeted therapy are constantly enhanced and created. At present, only surgical treatment is considered as a potential radical treatment for HCC. But only 15% of HCC patients have the opportunity to have surgery, most patients are found in the advanced stage (Roxburgh and Evans, 2008). Sorafenib is the only systemic medication approved by the FDA for advanced HCC. However, because of the overexpression of dihydropyrimine dehydrogenase, the multi-drug resistance gene MDR-1and p-glycoprotein gene products, HCC is regarded a chemotherapy-resistant tumour, and how to execute effective chemotherapy is still a major difficulty (Soini et al., 1996; Jiang et al., 1997; Kato et al., 2001).

Seven essential characteristics of cancer have been identified as crosstalk between cells and immune cells, self-sufficiency of signals for growth, unrestricted replication potential, apoptosis avoidance, growth signals insensitivity and continuous angiogenesis and invasion/metastasis of tissue (Hanahan and Weinberg, 2000; Hanahan and Weinberg, 2011). Tumor cells, immune cells, stromal cells, endothelial cells, and cancer-related fibroblasts are all found in the tumour microenvironment (TME), according to current research. Malignant tumour cells can evade immune monitoring and kill, as well as impair the human body, *via* a range of intricate ways (Hanahan and Weinberg, 2011). Due to the limitations of traditional chemotherapy regimens in the treatment of HCC, a variety of immunotherapy methods for HCC have been developed. Immunotherapy mostly employs immune cells within or outside of the TME to specifically target and assault cancer cells, with the benefits of high specificity and low side effects (Yost et al., 2019). More crucially, thanks to advances in tools such as mass spectrometry and single-cell RNA sequencing, We can map immunological cells in TMEs at the single-cell level (Spitzer and Nolan, 2016; Zheng et al., 2017; Papalexi and Satija, 2018; Wang et al., 2019a). We outline the significance of tumor-associated immune cells in the HCC tumour microenvironment and highlight their relevance in HCC cancer immunotherapy in this study.

## The Immune Cells in TME

**FIGURE 1:**
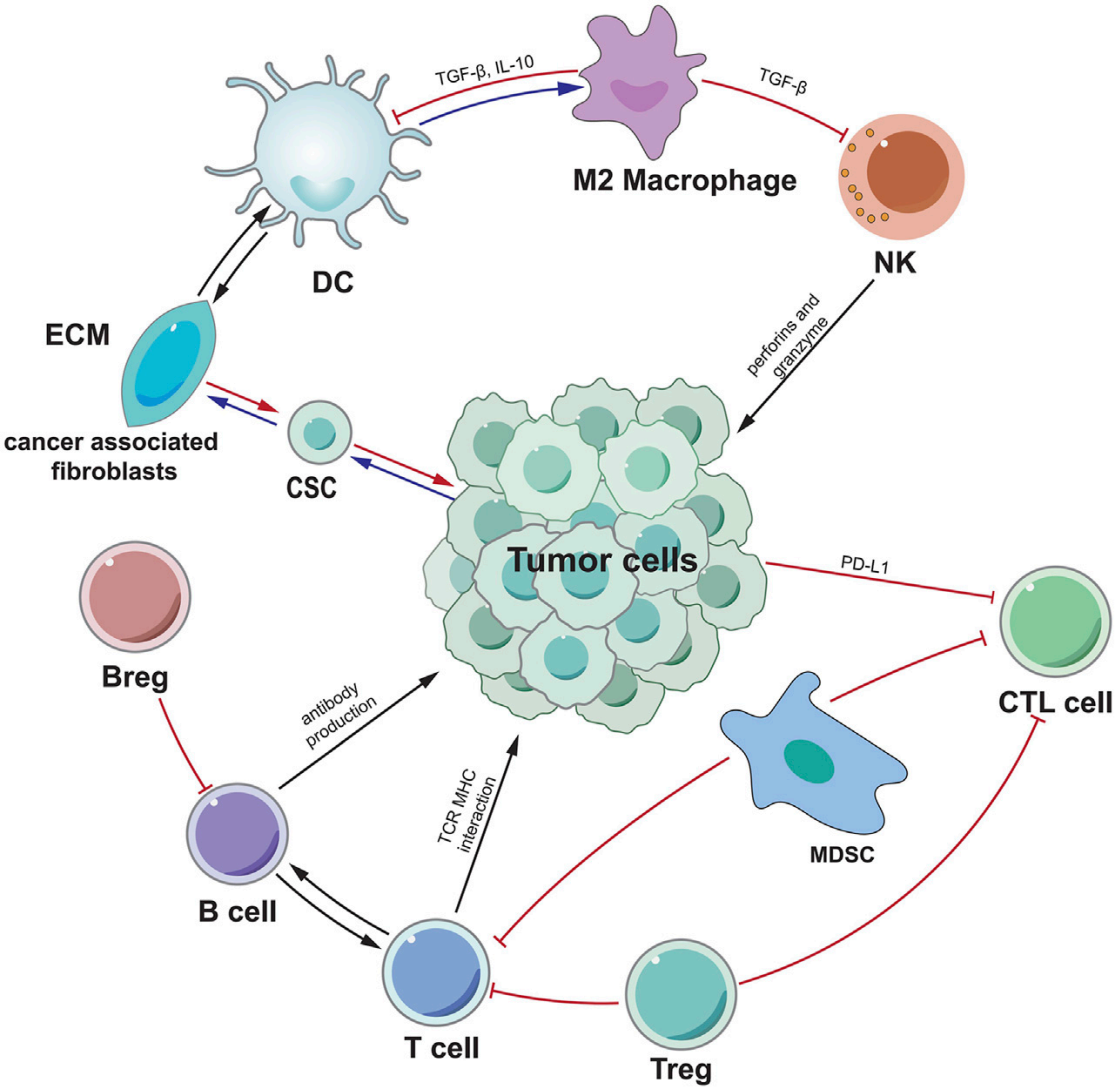
A schematic overview of the most important mechanisms and interactions of the tumor microenvironment. Tumor cells interact with other cells in various ways. CSC indicates cancer stem cell, ECM indicates extracellular matrix, CTL indicates cytotoxic T lymphocytes.

Tumor-associated immune cells are broadly classified into two types: tumor-promoting immune cells and tumor-antagonistic immune cells. At different stages of tumour formation, these two types of cells play different functions and impact each other (Figure 1). Because the significance of tumor-associated B cells in tumour growth is debatable, we shall introduce B cells additionally.

## Tumor-Antagonizing Immune Cells

### Effector T Cells

Current studies suggest that CD8^+^ cytotoxic T cells (CTLs) are the main lymphocytes that kill cancer cells. When CD8^+^ T cells recognize antibodies on DC, CD80^−^CD86 and CD70 ligands on DC connect to CD27 and CD28 receptors on CD8^+^ T cells, and CD8^+^ T cells are modified to become cytotoxic effector CD8^+^ T cells (Tanaka et al., 1999; Farhood et al., 2019). Furthermore, CD4^+^ T cells can activate CD8^+^ T cells through CD40^−^CD40L interaction, and CD4^+^ T cells can produce IL-2 to enhance CD8^+^ T cell proliferation. CD4^+^ T cells are also important in the maturation of CD8^+^ T cells into memory cells (Bennett et al., 1997; Mackey et al., 1997; Bennett et al., 1998; Mackey et al., 1998; Schoenberger et al., 1998; Bourgeois et al., 2002; Cheng et al., 2002; Borst et al., 2018). CTL kills target cells through granular exocytosis and apoptotic induction mediated by FasL ligand (FasL) in working state. CTL can also produce interferon- (IFN-) and tumour necrosis factor (TNF-) to cause cancer cell cytotoxicity (Farhood et al., 2019). Activation and regulation of CTL requires signals from T cell receptors (TCR) and immune checkpoints (Rogler et al., 1999). For example, cancer cells inhibit CTL activity through the expression of a ligand that binds to an inhibitory checkpoint, such as PD-L1 (Iwai et al., 2002). A significant number of studies have established the function of CD8^+^ T cells and CD4^+^ T cells in the formation and progression of HCC, including diagnosis/treatment/prognosis, and so on.

Chang et al. 's study confirmed that NanoMnSor improves the effectiveness of anti-PD-1 antibodies and whole-cell cancer vaccine immunotherapy by encouraging macrophage polarization to an immunostimulating M1 phenotype, decreasing hypoxica-induced tumor infiltration of tumor-associated macrophages, and raising the number of CD8 cytotoxic T cells in tumors, thereby reprogramming immunosuppressive TME (Chang et al., 2020). Xie et al. 's research proposed that PD-L1 overexpression is mostly triggered by pre-existing activated CD8 (+) cytotoxic T cells in the HCC environment, rather than being produced constitutively by tumor cells, and that it is a good prognostic factor for HCC (Xie et al., 2016). The frequency of circulating PD-1 (+) CD8 (+) T cells increases as the illness develops from LC to HCC. PD-1 expression was shown to be much higher in tumor-infiltrating CD8 (+) T cells. *In vitro*, CD8 (+) T cells promoted the production of PD-L1 on HCC cells in an IFN-dependent way, increasing CD8 (+) T cell death, whereas inhibiting PD-L1 reversed this effect (Shi et al., 2011).

Both *in vitro* restimulation and *in vivo* depletion studies have indicated that CD4^+^ and CD8^+^ lymphocytes contribute to anticancer activity. ASPH activation resulted in considerable production of antigen-specific CD4^+^ T cells in PBMC from healthy volunteers and HCC patients (Shimoda et al., 2012). Lack of recovered CD19^+^, CD3^+^, CD4^+^, and especially CD8^+^ T cells is associated with poor survival in patients (Carr and Metes, 2012). Zhou et al. ‘s revealed that antibodies against CD274 (PD-L1), LAG3, or TIM3 boost CD4^+^ and CD8^+^ TIL proliferation and cytokine secretion in response to polyclonal antigens or TAA stimulation (Zhou et al., 2017). More research results on the effect of Effector T cells in HCC are summarized in Table 1. It is clear that Effector T cells play a critical role in the immunological milieu of HCC. Many studies have shown that targeting these cells is effective in patients with HCC.

**TABLE 1:** The immune cells in TME: Effector T cells.

Cells	The research direction	Result	Reference
CD8^+^ T cell	The immune mechanism	NanoMnSor enhances the efficacy of anti-PD-1 antibodies and whole cell cancer vaccine immunotherapy by reducing tumor invasion of hypoxia-induced tumor-associated macrophages, promoting macrophage polarization into an immune-stimulating M1 phenotype and increasing the number of CD8 cytotoxic T to reprogram the immunosuppressive TME.	Chang et al. (2020)
CD8^+^ T cell	immunotherapy	PVRL1 upregulated by HCC cells stabilizes PVR on the cell surface, which interacts with the inhibitory molecule TIGIT of CD8 effector memory T cells. It is possible to develop PVRL1/TIGIT inhibitors as well as anti-PD1 to treat HCC.	Chiu et al. (2020)
CD8^+^ T cell	Prognostic marker	PD1 or TIM3PD1 CD8 T cells were significantly associated with poor prognosis, and the latter were adjacent to PD-L1 tumor-associated macrophages.	Ma et al. (2019)
CD8^+^ T cell	immunotherapy	SFGL2 promotes HCC growth by reducing DC activity and subsequent cytotoxicity of CD8 T cells, suggesting that SFGL2 is a novel potential therapeutic target for HCC therapy.	Yang et al. (2019a)
CD8^+^ T cell	The immune mechanism	IL-35 inhibits the cytolytic and noncytolytic functions of CD8 T cells against non-viral hepatitis associated HCC, possibly inhibiting the expression of perforin.	Yang et al. (2019b)
CD8^+^ T cell	The immune mechanism	Suppression of the tumor immunosuppressive environment and immune escape is accompanied by proliferation of functional cytotoxic CD8 T cells as well as suppression of myeloid suppressor cells and regulatory T cells in the tumor environment.	Tao et al. (2019)
CD8^+^ T cell	The immune mechanism	A large number of TEM-1 positive (TEM-1) TAM in the late stage of HCC development indirectly impair the cytotoxic function of CD8 T cells and induce the apoptosis of CD8 T cells.	Wu et al. (2019a)
CD8^+^ T cell	immunotherapy	The Δ- catenin peptide vaccine stimulates the activation of cytotoxic T lymphocytes (CTL) and enhances the invasion of CD8^+^ T cells into the tumor. In addition, Δ- catenin peptide vaccine can enhance the secretion of IFN-γ and the killing effect of T cells on tumor cells.	Huang et al. (2018)
CD8^+^ T cell	The immune mechanism	Stat3-blocked lysates of whole HCC cells stimulated activation of T and natural killer (NK) cells and enhanced cytotoxic CD8 T cell infiltration in tumor tissues.	Han et al. (2017)
CD8^+^ T cell	The immune mechanism	Antibody against CD274(PD-ligand 1 [PD-L1]), TIM3, or LAG3 increases CD8 and CD4 TIL proliferation and cytokine production in response to stimulation by polyclonal antigens or TAA.	Zhou et al. (2017)
CD8^+^ T cell	immunotherapy	Tremelimumab in combination with tumor ablation is a potential new therapy for patients with advanced HCC, resulting in the accumulation of CD8 T cells in tumors. Positive clinical activity was observed and may replace HCV viral load.	Duffy et al. (2017)
CD8^+^ T cell	immunotherapy	PD-L1 upregulation is mainly induced by pre-existing activated CD8^+^ cytotoxic T cells in the HCC environment, rather than constitutively expressed by tumor cells, and is a favorable prognostic factor for HCC.	Xie et al. (2016)
CD8^+^ T cell	The immune mechanism	Expression of programmed death 1(PD-1) and programmed death ligand 1(PD-L1) correlated with CD3^+^ and CD8^+^ cell density and clinical outcome. High density of internal and peripheral CD3^+^ and CD8^+^ T cells and corresponding immune scores were significantly associated with lower recurrence rates and prolonged RFS.	Gabrielson et al. (2016)
CD8^+^ T cell	Prognostic marker	Crosstalk of NK and CD8 T cells in tumor microenvironment may benefit patient prognosis. The count of NK and CD8 T cells infiltrating in CRC tumors may provide useful prognostic information	Sconocchia et al. (2014)
CD8^+^ T cell	immunotherapy	In the tumor microenvironment of sorafenib treated mice, tumor-specific effector T cells were upregulated, while the ratio of CD8^+^ T cells expressing PD-1 and regulatory T cells (Tregs) was reduced.	Chen et al. (2014)
CD8^+^ T cell	The immune mechanism	In HLA-A2 transgenic mice, the CD8^+^ T cells producing IFN-γ and the cytolytic activity *in vivo* were significantly increased.	Chen et al. (2012)
CD8^+^ T cell	The immune mechanism	As the disease progresses from LC to HCC, the frequency of circulating PD-1 (+) CD8^+^ T cells increases. Tumor-infiltrating CD8^+^ T cells showed a dramatic increase in PD-1 expression. *In vitro*, CD8^+^ T cells induced PD-L1 expression on HCC cells in an IFN-γ dependent manner, thereby promoting APOPTOSIS of CD8^+^ T cells, while blocking PD-L1 reversed this effect.	Shi et al. (2011)
CD8^+^ T cell	immunotherapy	Strong TAA-specific CD8^+^ T cell response inhibited HCC recurrence. For patients with HCC following local therapy, induction of TAA-specific cytotoxic T lymphocytes should be considered with immunotherapy, such as peptide vaccine.	Hiroishi et al. (2010)
CD8^+^ T cell	Prognostic marker	Overexpression of HLA-G protein in HCC is an independent indicator of poor prognosis, especially in early disease. The combination of HLA-G expression and Tregs/CD8+ ratio increased the prognostic power of both variables.	Cai et al. (2009)
CD8^+^ T cell	immunotherapy	HCA661 peptides H110 and H246 are naturally processed in dendritic cells (DCs) and when applied to DCs, they are sufficient to induce autologous CD8^+^ T cells to initiate cytotoxic responses against HCA661(+) human cancer cells.	Pang et al. (2007)
CD8^+^ T cell	Prognostic marker	The combination of low intratumoral Tregs with high intratumoral activated CD8^+^ cytotoxic cells (CTL) is the balance of CTL and is an independent prognostic factor for improved DFS and OS.	Gao et al. (2007)
CD8^+^ T cell	Prognostic marker	Increased CD4 (+) CD25 (+) Foxp3 (+) Treg may impair effector function of CD8^+^ T cells, promote disease progression, and represent a potential prognostic marker and therapeutic target in HBV-associated HCC patients.	Fu et al. (2007)
CD8^+^ T cell	immunotherapy	The functionally detectable presence of M3 (271) specific CD8^+^ T cells in HCC patients makes M3 (271) a potential target for immunotherapy in these patients. CD8^+^ T cells that respond to both NY-ESO-1 and MAG-A3 antigens provide a theoretical basis for the use of a bivalent vaccine in HCC patients with tumors expressing both antigens.	Zhang et al. (2007)
CD8^+^ T cell	Prognostic marker	HCC patients showed significantly higher WT p53-specific memory CD8^+^ T cell frequency and stronger WT p53-specific CTL activity compared to healthy controls. Increased frequency and activity of P53 specific CD8^+^ T cells were associated with deletion of selective HLA-A2 alleles and decreased expression of co-stimulatory molecules in tumor cells.	Cicinnati et al. (2006)
CD8^+^ T cell	The immune mechanism	CD4^+^ CD25 ^+^ T cells in the peripheral region of HCC may play a key role in controlling the activity of CD8^+^ cytotoxic T cells, thus promoting the development of HCC.	Yang et al. (2006)
CD4+T cell	The immune mechanism	The C/eBPα/miR-7 axis negatively regulates CD4+T cell activation and function through MAPK4, thus orchestrating experimental AIH mice.	Zhao et al. (2020)
CD4+T cell	The immune mechanism	*In vivo* treatment with MYC ASO without control ASO reduced proliferation, induced apoptosis, increased senescence, and remodeled the tumor microenvironment by recruiting CD4+T cells	Dhanasekaran et al. (2020)
CD4+T cell	The immune mechanism	Tumor associated CD4/CD8 double positive T (DPT) cells were found to be rich in L region, with co-expression of PD-1/HLA-DR/ICOS/CD45RO, and showed high levels of IFN-, TNF- and -1 after PD stimulation. Enrichment of DPT and PD-1DPT in L region indicated a good prognosis.	Zheng et al. (2020a)
CD4+T cell	The immune mechanism	The recruitment of cytotoxic cells, namely terminally differentiated CD4^+^ and CD8^+^ T cells (TEFF), is impaired in the tumor, and the effector memory CD4^+^ T cells are more attracted in this region.	Chaoul et al. (2020)
CD4+T cell	The immune mechanism	Knockdown of DCR3 expression in HCC significantly restored the immunity of CD4+T cells. Inhibition of DCR3 expression may provide a novel immunotherapy approach to restore immunity in HCC patients	Zhu et al. (2019)
CD4+T cell	The immune mechanism	Activated CD4+T cells from HCC stimulate macrophages to produce C-X-C motif chemokine 10(CXCL10). CXCL10 binds to the CXC chemokine receptor 3 on B cells and signals them through extracellular signal-regulated kinases, making them plasma cells that produce IgG.	Wei et al. (2019)
CD4+T cell	The immune mechanism	In a study of mice with liver tumors, CXCR6 was found to mediate the removal of NKT cells and CD4+T cells from senescent liver cells.	Mossanen et al. (2019)
CD4+T cell	immunotherapy	Liver-specific MYC transgenic mice fed the MCD diet were induced. Blockage of CPT with the pharmacological inhibitor perhexiline reduced apoptosis of CD4+T cells in the liver and inhibited tumor formation in HCC. These results provide useful information for the potential targeting of the CPT family to salvage intrahepatic CD4+T cells and for immunotherapy to assist NAFLD-promoted HCC.	Brown et al. (2018)
CD4+T cell	immunotherapy	Tumor-infiltrating LY6G MDSCs from orthotopic liver tumors treated with sorafenib significantly induced CD4+T cells expressing IL-10 and TGF-β and down-regulated the cytotoxic activity of CD8 T cells.	Chang et al. (2018)
CD4+T cell	The immune mechanism	Antibody against CD274(PD-ligand 1 [PD-L1]), TIM3, or LAG3 increases CD8 and CD4 TIL proliferation and cytokine production in response to stimulation by polyclonal antigens or TAA.	Zhou et al. (2017)
CD4+T cell	The immune mechanism	Blockage of PD-L1 can restore IFNγ/TNF-α production in BTLAPD-1 tumor CD4+T cells, but partially inhibit the activation of BTLAPD-1 CD4+T cells.	Zhao et al. (2016)
CD4+T cell	The immune mechanism	CB2 inactivation reduces expression of T cell recruitment chemokines and inhibits liver T cell recruitment, including specific CD4^+^ T cells.	Suk et al. (2016)
CD4+T cell	immunotherapy	IFNγ enzym-linked immunodot assay demonstrated that they induced antigenicity of specific CD4+T cells in healthy donors or in HCC patients before and after GPC3-SP vaccine administration. The natural processing of these epitopes was demonstrated by the immune response of helper T cells to dendritic cells (DCs) loaded with GPC3.	Sayem et al. (2016)
CD4+T cell	The immune mechanism	TGF-β expression was upregulated in DEN induced HCC mouse model. TGF-β promotes the differentiation of Foxp3 (+) CD4 (+) T cells (Treg cells) *in vitro*.	Shen et al. (2015)
CD4+T cell	immunotherapy	Intra-tumor combined administration of SLC and anti-CD25 mAb significantly reduced the frequency of Treg and increased the number of CD8^+^ and CD4^+^ T cells in tumor sites, and also maximally inhibited the growth and invasion of HCC.	Chen et al. (2013)
CD4+T cell	Prognostic marker	The high expression of IL-17 and IL-17RE is a promising predictor of poor prognosis in HCC. The precursor capacity of the CD4^+^ T cells that produce IL-17 may be involved in cross-talk of different types of inflammatory/immune cells than in HCC.	Liao et al. (2013)
CD4+T cell	The immune mechanism	CD25-Foxp3-T cells with a CD127-IL-10 + phenotype can be induced *in vitro* from naive CD4 (+) T cells involving programmed cell death 1 ligand 1, immunoglobulin-like transcript4, and human leukocyte antigen G.	Kakita et al. (2012)
CD4+T cell	The immune mechanism	*In vitro* restimulation experiments and *in vivo* depletion studies have shown that both CD4^+^ and CD8^+^ cells contribute to antitumor activity. Using PBMC from healthy volunteers and patients with HCC, it was shown that ASPH stimulation led to significant development of antigen-specific CD4^+^ T cells.	Shimoda et al. (2012)
CD4+T cell	Prognostic marker	Lack of recovered CD19, CD3, CD8, and especially CD4+T cells is associated with poor survival in patients.	Carr and Metes, (2012)
CD4+T cell	immunotherapy	Tregs significantly increased the inhibition of CD8^+^ and CD4 (+) T cell proliferation and cytokine secretion, and increased numbers of circulating CD4 (+) CD25 (+) Foxp3 (+) Tregs and tumor-infiltrating Foxp3 (+) cells prior to cryoablation were associated with a higher risk of recurrence or progression in HCC patients after cryoablation.	Zhou et al. (2010)
CD4+T cell	The immune mechanism	MDSC exerts its immunosuppressive function by inducing CD4 (+) CD25 (+) Foxp3 (+) regulatory T cells in co-cultured CD4 (+) T cells.	Hoechst et al. (2008)
CD4+T cell	immunotherapy	CD4^+^ T cells killed gene-independent CT26 cells and even homologous HEPA1-6 cells. In mice treated with DC/BNL + IL-12, a large number of CD4^+^ T cells and MHC class II positive macrophages infiltrated the tumor tissue.	Homma et al. (2005)
CD4+T cell	The immune mechanism	TRAIL receptors on HCC cells were upregulated by 5-FU, and TRAIL on CD4 (+) T cells, CD14 (+) monocytes and CD56 (+) NK cells was induced by IFNα.	Yamamoto et al. (2004)
CD4+T cell	The immune mechanism	IFN-γ was produced by incubation with DC/MIH-2 from CD4 (+) T cells but not from CD8^+^ T cells of inoculated mice. Anti-IFN-γ antibody attenuated the cytotoxicity of spleen cells. Immunization of CD4 (+) T cells with DCs loaded with homologous HCC cells, which produce IFN-γ in response to HCC antigens, leads to activation of macrophages that kill liver tumor cells at an early stage.	Irie et al. (2004)

### NK Cells

NK cells are an essential anti-tumor immune cell that primarily mediates immune surveillance of malignancies. It performs a similar function as CD8^+^ T cells: NK cells regulate the killing response of tumor cells by releasing perforin and granulein, triggering apoptosis in target cells. In addition, to improve their anticancer activity, NK cells can produce proinflammatory cytokines and chemokines (Voskoboinik et al., 2006; Guillerey et al., 2016; Habif et al., 2019). Existing studies have confirmed the value of NK cells in the development, targeted therapy, prognosis of HCC. Sprinzl et al. 's research confirmed that Sorafenib can promote the pro-inflammatory response of tumor-associated macrophages in HCC, and then activate the anti-tumor NK cell response through the cytokine and NF-κB pathway (Sprinzl et al., 2013). Senescence monitoring necessitates the recruitment and maturation of CCR2 myeloid cells, and CCR2 deficiency promotes HCC growth. Conversely, HCC cells suppress the maturation of recruited myeloid progenitors, which promotes mice HCC growth and worsens prognosis and survival in human HCC patients *via* NK cell inhibition (Eggert et al., 2016). Kohga et al. 's revealed that natural killer (NK) cells had stronger cytolytic activity on ADAM9KD-HCC cells than on control cells, and that this cytotoxicity is enhanced by the MICA/B and NK group 2, D pathways. Sorafenib treatment resulted in a decrease in ADAM9 expression in HCC cells, an increase in membrane-bound MICA expression, and a decrease in the quantity of soluble MICA. Sorafenib increased HCC cell NK sensitivity by boosting the expression of membrane-bound MICA (Kohga et al., 2010a). Table 2 summarizes current research on NK cells in HCC, confirming the importance of NK cells in immune escape and anti-HCC therapy.

**TABLE 2:** The immune cells in TME: NK cells.

Cells	The research direction	Result	Reference
NK cells	immunotherapy	Sorafenib can promote the pro-inflammatory response of tumor-associated macrophages in HCC, and then activate the anti-tumor NK cell response through the cytokine and NF-κB pathway.	Sprinzl et al. (2013)
NK cells	The immune mechanism	NK cell activator Poly (I:C) promotes HCC in HBs-Tg mice. Poly (I:C) induces liver inflammation and liver cell damage in HBs-Tg mice. The increase of hepatocyte EMT depends on the presence of NK cells in HBs-Tg mice. IFN-γ derived from NK cells plays a key role in the development of HCC in HBs-Tg mice.	Chen et al. (2019)
NK cells	The immune mechanism	The phenotype of peripheral blood NK cells was biased towards the defect/fatigue immune pattern, and the frequency of cells expressing NKp30 and member D of natural killer group 2 decreased, and the proportion of cells expressing T cell immunoglobulin and mucin domain increased. In addition, nKP30-positive NK cells have reduced expression of NCR3 immunostimulated splicing variants and increased expression of inhibitory variants, leading to NKP30-mediated loss of function in patients with advanced tumors.	Mantovani et al. (2019)
NK cells	Prognostic marker	Blocking the CD96^−^CD155 interaction restores NK cell immunity to tumor by reversing NK cell depletion or TGF-β1 reversing NK cell depletion, suggesting that CD96 may have a therapeutic role in HCC.	Sun et al. (2019a)
NK cells	The immune mechanism	When co-cultured with sorafenib treated M φ, cytotoxic NK cells were activated, resulting in tumor cell death. In addition, sorafenib was found to down-regulate the expression of major histocompatibility complex I in tumor cells, which may reduce tumor response to immune checkpoint therapy and promote NK cell response.	Hage et al. (2019)
NK cells	immunotherapy	Serum cholesterol accumulates in NK cells and activates their effect on HCC cells, increases the anti-tumor function of natural killer cells, and reduces the growth of liver tumors in mice.	Qin et al. (2020)
NK cells	The immune mechanism	CD48 protein was strongly expressed in HCC tissues but not in tumor liver monocytes. This monocyte induced NK cell dysfunction was significantly attenuated by blocking CD48 receptor 2B4 on NK cells, but not by blocking NKG2D and NKp30.	Wu et al. (2013)
NK cells	The immune mechanism	The cytotoxicity of NK cells in patients with HCC is reduced. MDSCs inhibit NK cell cytotoxicity and IFN-γ release. MDSCs inhibit NK cells depending on cell contact. MDSCs inhibit NK cells for a long time. MDSCs use NKp30 receptors to inhibit NK cell function.	Hoechst et al. (2009)
NK cells	The immune mechanism	Compared with donor and recipient PB, donor liver NK cells showed the strongest cytotoxicity to HCC HepG2 after IL-2 stimulation. This may explain why liver natural killer (NK) cells have higher cytotoxic activity against tumor cells than peripheral blood (PB) NK cells.	Ishiyama et al. (2006)
NK cells	The immune mechanism	SMICA derived from advanced HCC is responsible for NKG2D expression and NK cell function. NK cells stimulate DC maturation induced by human hepatoma cells and enhance the excitatory stimulation ability of DC. When NK cells were pretreated with serum containing SMicas, DC maturation and activation were completely eliminated.	Jinushi et al. (2005)
NK cells	immunotherapy	Leptin can significantly inhibit human HCC. This effect is mediated by inducing the proliferation and activation of natural killer cells and directly inhibiting tumor growth. The decreased NK expression of inhibitory CIS and the overexpression of anti-proliferative STAT2 and SOCS1 proteins in HCC lines may emphasize the anticancer effect of leptin.	Elinav et al. (2006)
NK cells	The immune mechanism	Smoking is associated with a decrease in the frequency of natural killer (NK) cells in the peripheral blood, which is characterized by a reduction in NK function through systemic immunology. The combination of smoking and lowering the frequency of NK cells further increases the likelihood of viral load and ALT≥80 U/L.	Wang et al. (2019b)
NK cells	The immune mechanism	NKT and CD4+T cells promote the elimination of senescent liver cells to prevent the occurrence of HCC, and this process requires CXCR6. CXCR6 inhibits the occurrence of HCC by promoting natural killer T and CD4+T cell-dependent senescence control.	Mossanen et al. (2019)
NK cells	immunotherapy	Using CAR transduced T cells and NK cells that recognize the surface marker CD147 (also known as Basigin), various malignant HCC cell lines were effectively killed *in vitro*, as well as HCC tumors in transplanted and patient-derived mouse models of transplanted tumors.	Tseng et al. (2020)
NK cells	The immune mechanism	The liver gene delivery of high IL-15 makes CD8^+^ T cells and NK cells proliferate in large quantities, resulting in the accumulation of CD8^+^ T cells in the body (over 40 days), especially in the liver. Hyper-IL-15 therapy has significant therapeutic effects on established liver metastases and even autologous HCC induced by DEN. These effects can be depleted by CD8^+^ T cells instead of NK cells.	Cheng et al. (2014)
NK cells	immunotherapy	The cytolytic activity of natural killer (NK) cells on ADAM9KD-HCC cells is higher than that on control cells, and the enhancement of this cytotoxicity depends on the MICA/B and NK group 2, D pathways. Sorafenib treatment resulted in a decrease in the expression of ADAM9 in HCC cells, an increase in the expression of membrane-bound MICA and a decrease in the level of soluble MICA. The addition of sorafenib enhanced the NK sensitivity of HCC cells by increasing the expression of membrane-bound MICA.	Kohga et al. (2010a)
NK cells	The immune mechanism	ADAM9 protease plays a key role in the shedding of MHC class I related chain A (MICA) that regulates the sensitivity of tumor cells to natural killer cells (NK). The expression of ADAM9 in CD133si-PLC/PRF/5 cells and CD133-Huh7 cells decreased, membrane-bound MICA increased, and soluble MICA production decreased. CD133si-PLC/PRF/5 cells and CD133-Huh7 cells are both sensitive to NK activity, which depends on the expression level of membrane-bound MICA, while HCC cells expressing CD133 are not.	Kohga et al. (2010b)
NK cells	The immune mechanism	CD8^+^ T cells and NKT cells promote NASH and HCC by interacting with hepatocytes, but not myeloid cells. NKT cells mainly cause steatosis by secreting light, and CD8^+^ and NKT cells synergistically induce liver damage. Hepatocyte LTβR and typical NF-κB signals promote the transformation of NASH to HCC, indicating that different molecular mechanisms determine the development of NASH and HCC.	Wolf et al. (2014)
NK cells	The immune mechanism	In-depth studies of the immune landscape show that regulatory T cells(T) and CD8 resident memory T cells(T) are enriched in hbv-related HCC, while Tim-3CD8 T cells and CD244 natural killer cells are in non-virus-related HCC In the enrichment.	Lim et al. (2019)
NK cells	The immune mechanism	Senescence monitoring requires the recruitment and maturation of CCR2 bone marrow cells, while CCR2 ablation induces HCC growth. In contrast, HCC cells inhibit the maturation of recruited myeloid precursors, which promote mouse HCC growth and deteriorate the prognosis and survival of human HCC patients by inhibiting NK cells.	Eggert et al. (2016)
NK cells	The immune mechanism	β-glucosylceramide alleviates immunologically incongruent disease by altering the plasticity of NKT lymphocytes and may be involved in the “fine-tuning” of the immune response.	Zigmond et al. (2007)
NK cells	The immune mechanism	Proliferating immune cells, mainly NK cells and T cells, were present in the patient’s long-lived tumor and consisted only of tumor cells lacking proliferation in the region. The density of NK cells and CD8+T cells was positively correlated with tumor cell apoptosis and negative proliferation.	Chew et al. (2010)
NK cells	immunotherapy	GS-9620 treatment is associated with a reversible increase in serum liver enzymes and thrombocytopenia, and induction of intrahepatic CD8^+^ T cells, NK cells, B cells, and interferon response transcriptional signaling.	Menne et al. (2015)
NK cells	The immune mechanism	Fibrosis is a way to enhance the occurrence of HCC. Changes in fibrosis also regulate the activity of inflammatory cells in the liver and reduce natural killing, which is generally helpful for tumors to monitor the activity of natural killer T cells. These pathways work in conjunction with inflammatory signals, including telomerase activation and the release of reactive oxygen species, ultimately leading to cancer.	Zhang and Friedman, (2012)
NK cells	immunotherapy	AdCMVmCD40L therapy can induce strong lymphocyte infiltration in tumor tissues and increase the apoptosis of malignant cells. The observed anti-tumor effect is mediated by CD8^+^ T cells and is related to increased serum levels of interleukin (IL)-12 and enhanced natural killer (NK) activity.	Schmitz et al. (2001)
NK cells	The immune mechanism	The activation of β-catenin in hepatocytes can change the liver microenvironment and lead to the specific targeting of iNKT cells. The activation of β-catenin in hepatocytes triggers the pro-inflammatory process related to the activation of NF- κ B. In the process of liver inflammation induced by β-catenin, iNKT cells showed anti-inflammatory properties. In HCC induced by β-catenin, iNKTs and LECT2 are the key cellular and molecular effectors to control tumor progression.	Anson et al. (2012)
NK cells	The immune mechanism	AdCMVIL-12 can activate natural killer cells (NK) and inhibit angiogenesis.	Barajas et al. (2001)
NK cells	The immune mechanism	Gene transfer of angiostatin inhibited tumor angiogenesis and enhanced NK cell infiltration, while B7H3 therapy activated CD8^+^ and NK cells and increased their infiltration into the tumor, and enhanced circulating IFN-γ levels.	Ma et al. (2007)

### Dendritic cells

DC cells, as specialized antigen-presenting cells in the human body, can present antigens to T cells and produce costimulatory signals for T cell activation. According to the current study, mature DC cells can penetrate tumor cells and limit tumor incidence and progression. Under many severe conditions, this inhibition effect will be avoided by tumors through certain means. Therefore, targeting at DC cells, some studies have reported its role in the occurrence, development, immunotherapy, diagnosis and prognosis of HCC. For example, In mice, combining DC vaccination and PD-L1 inhibitor treatment can result in longer overall life, reduced tumor volume, and increased tumor cell apoptosis. As a new therapy method for HCC, combined treatment with DC vaccination and PD-L1 inhibitor may offer promising results (Teng et al., 2020). Ali et al. 's research clarified that the combination of PEI or RFTA with active antigen-specific immunotherapy using DCS is a promising approach to induce a sustained anti-tumor immune response aimed at reducing tumor recurrence and metastasis in patients with HCC. Table 3 summarizes the current role of DC cells in HCC.

**TABLE 3:** The immune cells in TME: Dendritic cells.

Cells	The research direction	Result	Reference
Dendritic cells	immunotherapy	The combination of DC vaccine and PD-L1 inhibitor resulted in longer overall survival, smaller tumor size, and higher tumor cell apoptosis rate in mice. The combination of DC vaccine and PD-L1 inhibitor may have broad prospects as a new therapeutic strategy for HCC.	Teng et al. (2020)
Dendritic cells	The immune mechanism	XCL1-GPC3 cells chemically attract murine XCR1CD8α dendritic cells (DCs) and human XCR1CD141 DCs, and promote their IL12 production.	Chen et al. (2020)
Dendritic cells	The immune mechanism	A more severe reduction in basal OCR was observed in tumor-derived DCs exposed to AFP due to down-regulation of cytochrome C oxidase. The expression of PGC1-α in circulating medullary DC in HCC patients was decreased, and the ability to stimulate the function of antigen-specific effector was impaired, indicating the negative effect of AFP on DC metabolism.	Santos et al. (2019)
Dendritic cells	The immune mechanism	AFP down-regulated CD1 on DC, but had little effect on NKT cell activation.	Li et al. (2019a)
Dendritic cells	The immune mechanism	DC-CIK cells can inhibit the growth of HCC and LCSC *in vitro* and *in vivo*, and the most successful DC-triggered cell killing activity can be achieved through their LCSC antigen loading.	Yang et al. (2018b)
Dendritic cells	The immune mechanism	MIP-10 enhances the antitumor activity of the DC/tumor fusion vaccine by alleviating the immunosuppressive tumor environment.	Hu et al. (2017)
Dendritic cells	immunotherapy	The use of nifurazine and DC-loaded TCL significantly increased the survival rate, inhibited tumor growth and promoted anti-tumor immune response in HCC mice implanted *in situ*.	Zhao et al. (2017b)
Dendritic cells	immunotherapy	Overexpression of IL-12 induced by adenovirus vector can effectively immunize DC. Intratumor but not systemic injection of activated IL-12-DC is essential for effective tumor regression. Improved immunotherapy with IL-12-DC represents a promising approach for the treatment of HCC.	Vogt et al. (2014)
Dendritic cells	immunotherapy	β-GC activated mouse liver NKT cells and enhanced the anti-tumor activity of PAD-HBsAg-DC. β-GC may act as an effective innate immune enhancer to enhance the antitumor effect of PAD-HBsAg-DC vaccine.	Long et al. (2013)
Dendritic cells	immunotherapy	Camory may play an important role in the development and progression of HCC through recruitment of DC and NK cells.	Lin et al. (2011)
Dendritic cells	The immune mechanism	Electroporation of GPC-3 mRNA is an effective method for antigen-carrying monocytes to generate DCs because they produce functional GPC-3 reactive T cells *in vitro.*	O'Beirne et al. (2010)
Dendritic cells	The immune mechanism	Immature DCs(IMDCs) derived from human monocytes were fully mature after effective phagocytosis of dying cells, and showed significant proliferation and cytotoxicity to HLA-matched HEPG (2) cells in autologous peripheral blood monocytes (PBMC).	Xing et al. (2009)
Dendritic cells	The immune mechanism	Secondary lymphoid tissue chemokines (SLC) are strongly expressed in secondary lymphoid organs. It has the ability to promote dendritic cell (DC) and T cell chemotaxis, making it a promising candidate for cancer therapy.	Liang et al. (2007)
Dendritic cells	immunotherapy	AdvHAFP-transduced DCs activate higher frequencies of Th1 CD4 responses to AFP in healthy donors and AFP positive HCC patients. Importantly, when activated by adenovirus-engineered DC, the cytokine expression profile of CD4+T cells was biased towards the production of interleukin-2 and interferon-γ, which has therapeutic implications for vaccination work.	Evdokimova et al. (2007)
Dendritic cells	immunotherapy	Dendritic cells transfected with total HCC mRNA stimulated an antigen-specific cytotoxic T cell response capable of recognizing and killing autologous tumor cells *in vitro*.	Peng et al. (2006)
Dendritic cells	The immune mechanism	Impairment of MDCs produced by IL-12 may result in impaired stimulation of naive T cells, suggesting that targeted IL-12 therapy may enhance tumor-specific immune responses in patients with HCC.	Ormandy et al. (2006)
Dendritic cells	immunotherapy	The combination of PEI or RFTA with active antigen-specific immunotherapy using DCS is a promising approach to induce a sustained anti-tumor immune response aimed at reducing tumor recurrence and metastasis in patients with HCC.	Ali et al. (2005)
Dendritic cells	The immune mechanism	The phenotypic and functional deficits of PBMC-derived DCs in LC and HCC patients may play a key role in HBV infection and HCC immune escape. After tumor is stimulated by antigen, DC function can be enhanced in LC patients.	Wong et al. (2005)
Dendritic cells	The immune mechanism	Ad-HBsAg-transduced DCs stimulated a strong cytotoxic T lymphocyte (CTL) response to HBsAg-expressing tumor cells and protected mice from lethal tumor attack.	Qiu et al. (2005)
Dendritic cells	immunotherapy	At concentrations up to 20 μg/mL, AFP did not alter the *in vitro* generation, maturation and T cell stimulation of DC. Higher AFP concentration ( >20 μg/ml) led to phenotypic changes in DC without impair its ability to stimulate CD4^+^ T cells. Independent of serum AFP levels, the frequency and function of DC and AFP-specific T cells did not decrease in HCC patients.	Ritter et al. (2004)
Dendritic cells	immunotherapy	Autologous DCs containing the HCA587 protein can induce specific T cell responses in healthy individuals by *in vitro* stimulation, and HCA587 is a good candidate for the development of a protein-based therapeutic DC tumor vaccine for the treatment of HCC patients.	Li et al. (2004)
Dendritic cells	immunotherapy	DCs transfected with MAGE-1 gene can induce higher cytotoxicity of SMMC7721 *in vitro*, suggesting that this transgenic DC may have the potential to induce specific antitumor activity and be used as a novel HCC vaccine.	Liu et al. (2001)
Dendritic cells	The immune mechanism	Decline in DC function and normal T cell response were observed in HBV-infected HCC patients, suggesting that the low function of DC is related to the pathogenesis of HBV or HCV-infected HCC.	Kakumu et al. (2000)

### M1-Polarized Macrophages

**FIGURE 2:**
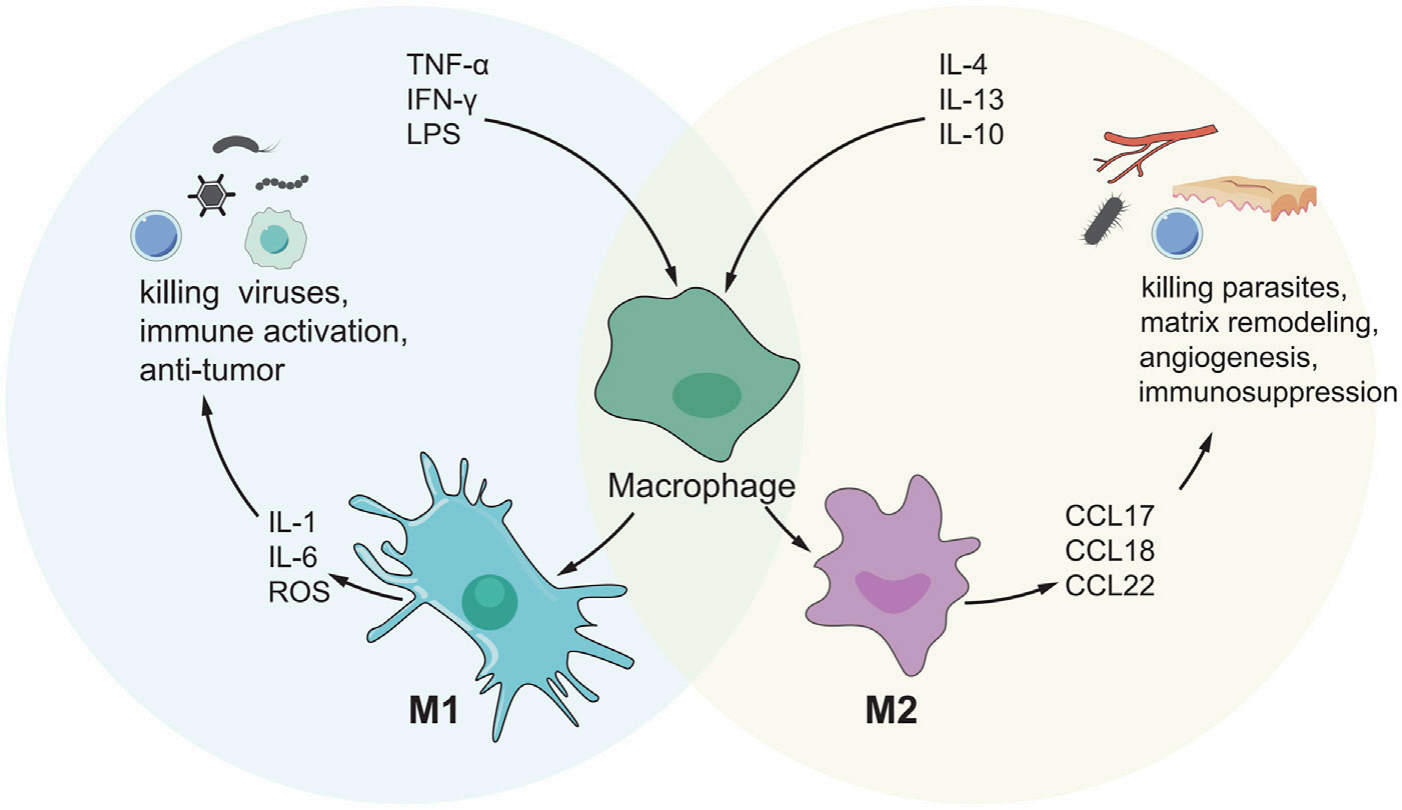
M1/M2 model of macrophage activation. M1 cells exert an inflammatory phenotype and are involved in killing bacteria, viruses and tumor cells, while M2 cells are involved in killing encapsulated parasites, immunosuppression, angiogenesis, etc.

Another important type of immune cell in TME is macrophages derived from circulating monocytes, which can generally be divided into M2 polarization and M1 polarization (Figure 2). M1-polarized macrophages, for example, can create pro-inflammatory cytokines and reactive oxygen species/nitrogen to prevent the formation and progression of malignancies (Aras and Zaidi, 2017). There are limited investigations on the function of M1-polarized macrophages in HCC at the moment. Studies have shown that M1-polarized macrophages in the S3 subclass in HCC increased, and the prognosis was good. Memory B cells, Total B cells, M1 macrophages and T follicle helper cells, were linked with strong total immune cell infiltration into HCC, whereas resting mast cells, neutrophils, and NK cells were associated with poor infiltration (Rohr-Udilova et al., 2018). Table 4 summarizes the current role of M1-polarized macrophages in HCC.

**TABLE 4:** The immune cells in TME: M1-polarized macrophages.

Cells	The research direction	Result	Reference
M1-polarized macrophages	The immune mechanism	The number of M1-polarized macrophages in the S3 subclass in HCC was increased, and the prognosis was good. Strong total immune cell infiltration into HCC was associated with total B cells, memory B cells, T follicle helper cells, and M1 macrophages, while weak infiltration was associated with resting NK cells, neutrophils, and resting mast cells.	Rohr-Udilova et al. (2018)
M1-polarized macrophages	The immune mechanism	Conditionalized media from M1 macrophages promoted HCC cell migration and induced activation of NF-κB and FAK signal transduction. Activation of Bay 11-7,802 and NF-κB and FAK pathways eliminated HCC cell induced migration, suggesting that M1-like TAM has a metastatic role in HCC.	Wang et al. (2014)

## Tumor-Promoting Immune Cells

In the immune microenvironment of HCC, some cells could promote the occurrence and development of HCC, and we will review them one by one.

### Regulatory T cells(Tregs)

Tregs play a vital role in immunological homeostasis and immune self-tolerance, and they can express the CD4^+^ marker and the Foxp3 marker (Samstein et al., 2012). Foxp3+ Treg acts as a switch for all levels of immune response, and its effects appear to be two-sided. First, Treg can inhibit harmful immune responses and thus inhibit the occurrence of autoimmune diseases (Berod et al., 2012). Second, Treg suppresses protective immune responses against invading pathogens or tumors, leading to further progression of the disease (Sakaguchi et al., 2010). How does Treg play a role in tumors, including how does Treg infiltrate and metastasize to tumor sites or how does Treg help tumors evade immune monitoring, has become a hot research topic in recent years. Many ideas have been put forward. Tregs were found in much higher numbers in HCC patients than in healthy controls. In addition, patients with high Treg(III) levels after TACE had a significantly lower progression-free survival than patients with low Treg(III) levels after TACE (Park et al., 2020). Other studies have shown that tumor Treg upregulated the expression of the glucocorticoid-induced tumor necrosis factor receptor (GITR). Treatment with soluble GITR ligand (GITRL) reduced inhibition caused by activated tumor infiltrating Treg and restored CD4^+^ CD25-T cell proliferation and cytokine production. (Pedroza-Gonzalez et al., 2013). In addition to that, the proportion of Tregs cells in HCC patients was significantly higher than that in healthy and cirrhosis controls, and was related to various clinical indicators of HCC patients. In HCC patients with BCLC stage C, the proportion of Treg cells was more pronounced than in BCLC stage B patients. One to 2 weeks after surgery, the fraction of Treg cells was much lower than before GSMS-TACE. Three to 5 weeks following surgery, the proportion of Treg cells continued to decline (Ren et al., 2021). Table 5 summarizes the most credible studies related to the role of Treg in HCC.

**TABLE 5:** Tumor-promoting immune cells: Regulatory T cells.

Cells	The research direction	Result	Reference
Treg	immunotherapy	The proportion of Treg cells in BCLC stage C HCC patients was higher than that in BCLC Stage B patients, and the proportion of Treg cells was significantly lower than that before GMS-TACE 1 ∼ 2 weeks after surgery. The percentage of Treg cells continued to decrease 3–5 weeks after surgery.	Ren et al. (2021)
Treg	The immune mechanism	Tumor progression is associated with the deep depletion of tumor antigen-specific CD8T cells and the accumulation of PD-1 CD8T cells and Treg cells.	Tipton et al. (2011)
Treg	The immune mechanism	At a non-cytotoxic concentration, resveratrol inhibited differentiation of CD8CD122 Treg from CD8CD122 T cells.	Zhang et al. (2020)
Treg	immunotherapy	The baseline reduction in the number or frequency of FOXP3Tregs, MDSCs, and exhausted T cells was significantly greater after tivozanib treatment. In addition, a larger increase in CD4T cells: Treg ratio after tivozanib treatment compared with sorafenib was associated with a significant improvement in OS.	Kalathil et al. (2020)
Treg	The immune mechanism	Long-term intake of ethanol can enrich HBV-enhanced abnormal lipid metabolism through HBx/Swaell1/arachidonic acid signaling and activate Treg in mice.	Liu et al. (2020a)
Treg	The immune mechanism	Lower CD8 T cell density and higher Foxp3 Tregs and immune checkpoint strength in intrahepatic cholangiocarcinoma (ICC) components compared to HCC components may indicate a stronger immune escape capability of ICC.	Zheng et al. (2020b)
Treg	Prognostic marker	The frequency of Tregs was significantly higher in HCC patients than in healthy controls. In addition, patients with high Treg (III) after TACE had a significantly reduced progression-free survival compared to patients with low Treg (III) after TACE.	Park et al. (2020)
Treg	The immune mechanism	Regulatory CD4/CD25/Foxp3 T cells (Tregs) were activated by transcriptional reprogramming of HCC parent cells in MacroH2A1 KD conditioned medium. Loss of MacroH2A1 in HCC cells drives proliferation and avoidance of immune surveillance by cancer stem cells.	Lo Re et al. (2020)
Treg	The immune mechanism	Treg-induced inhibition of IFN-γ secretion is partially prevented by neutralizing PD-1 and PD-L1 antibodies in HCC patients. In HCC, peripheral blood Tregs upregulate checkpoint inhibitors and promote systemic immune dysfunction and antitumor activity through several inhibitory pathways, presumably contributing to the development of tumors at a young age.	Langhans et al. (2019)
Treg	Prognostic marker	Patients with an increased T-effector/Treg ratio before treatment showed significant improvement in OS.	Kalathil et al. (2019)
Treg	The immune mechanism	Overexpression of lncRNA FENDRR and down-regulated miR-423-5p reduced cell proliferation and tumorigenicity, and promoted apoptosis of HCC cells, thereby regulating Treg-mediated immune escape of HCC.	Yu et al. (2019a)
Treg	immunotherapy	The hypoxic environment induces tumor immunosuppression by attracting TREM-1 TAMs of CCR6. Foxp3 Treg, and TREM-1 TAMs make HCC resistant to PD-L1 therapy	Wu et al. (2019a)
Treg	immunotherapy	After sorafenib alone and in combination, plasma SMet was elevated and TTP and OS shortening were associated with increased Tregs and CD56 natural killer (NK) cells.	Goyal et al. (2019)
Treg	immunotherapy	Sunitinib treatment induces an anti-tumor immune response by significantly reducing Treg frequency, TGF-β and IL-10 production by Treg, and protecting TAS CD8 T cells from HCC infection	Liu et al. (2017)
Treg	immunotherapy	Newly produced STAT3-blocked whole-cell HCC vaccines reduced production of Treg as well as production of TGF-β and IL-10.	Han et al. (2017)
Treg	The immune mechanism	LNC-EGFR specifically binds to EGFR and prevents its interaction with c-CBL and is ubiquitinated by c-CBL, which stabilizes and enhances the activation of its own and its downstream AP-1/NF-AT1 axis, thus causing EGFR expression. LNC-EGFR has been associated with immunosuppressive status and cancer by promoting Treg cell differentiation.	Jiang et al. (2017b)
Treg	The immune mechanism	Overexpression and activation of YAP-1 in HCC T cells can promote differentiation of Treg through enhanced transcription of TGFbR2, thereby inducing immunosuppression.	Fan et al. (2017)
Treg	Prognostic marker	CXCL10/CXCR3 signals were upregulated after liver graft injury, directly inducing the mobilization and recruitment of Tregs during transplantation, and further promoting the recurrence of HCC after transplantation.	Li et al. (2016)
Treg	immunotherapy	After treatment with sorafenib, the ratio of CD4CD127PD-1 T effector cells to CD4FoxP3PD-1 Treg was significantly increased. The increased frequency of CD4CD127 T effector cells in posttreated samples was significantly correlated with OS.	Kalathil et al. (2016)
Treg	immunotherapy	Sunitinib-mediated tumoricidal effect, Treg inhibition and antibody-mediated PD-1 blocking synergistic effect can effectively inhibit tumor growth and activate anti-tumor immunity.	Li et al. (2017)
Treg	The immune mechanism	The secreting of cancerous TGF-β1 may increase Tregs, while TGF-β1 knockdown may impair immunosuppression in the tumor microenvironment by decreasing Tregs.	Wang et al. (2016)
Treg	The immune mechanism	Intra-tumor combination of SLC and anti-CD25 mAb is an effective method for the treatment of HCC, which is related to the change of tumor microenvironment and the systematic optimization percentage of Treg, CD8^+^ T cells and CD4^+^ T cells in peripheral immune organs.	Chen et al. (2013)
Treg	immunotherapy	Sorafenib treatment can reduce the number of Treg by inhibiting the proliferation of Treg and inducing its apoptosis. In addition, sorafenib inhibits the function of Tregs, characterized by reduced expression of functionally important Treg-related molecules and impaired inhibition. Sorafenib treatment alleviated non-cellular autonomous inhibition of the tumor microenvironment mediated by Treg, leading to an effective anti-tumor immune response.	Chen et al. (2014)
Treg	immunotherapy	The subpharmacological doses of sorafenib had different effects on T cell subsets, selectively increasing Teff activation while blocking Treg function.	Cabrera et al. (2013)
Treg	The immune mechanism	Tumor Treg upregulated glucocorticoid-induced tumor necrosis factor receptor (GITR) expression. Treatment with soluble GITR ligand (GITRL) induced a reduction of inhibition mediated by activated tumor infiltrating Treg and restored the proliferation and cytokine production of CD4^+^ CD25-T cells.	Pedroza-Gonzalez et al. (2013)
Treg	The prognosis	Elevated FOXP3 (+) Tregs may represent a prognostic marker in patients with early HCC. The natural history of CHB influences the density of tumor infiltrate Treg in patients with chronic hepatitis B virus infection.	Wang et al. (2012)
Treg	The immune mechanism	The increased frequency of CD45Ro + subsets in CD4^+^ CD25(HIGH) Tregs in HCC patients may cooperate with the establishment of an immunosuppressive environment that induces tolerance of plasmocyte like DCs, thereby promoting the development of HCC.	Takata et al. (2011)
Treg	The immune mechanism	The tumor-associated MVARPHI may trigger an increase in the number of Foxp3 (+) Treg populations in tumors, thereby promoting the development of HCC.	Zhou et al. (2009)

### Myeloid-Derived Suppressor cells

**FIGURE 3:**
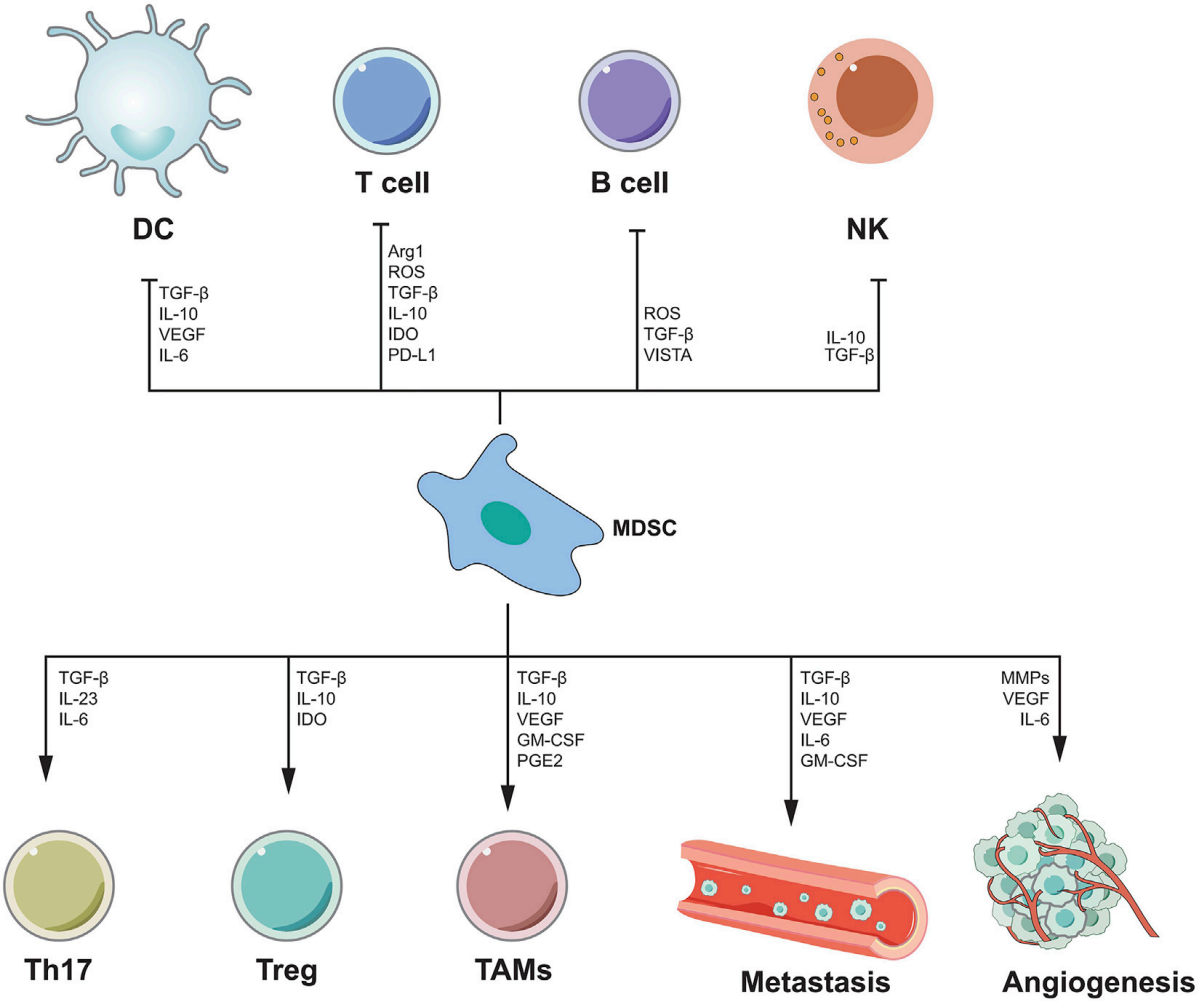
The mechanism of MDSC-mediated immunosuppression. MDSC inhibits the functions of DC, T cells, B cells and NK cells by secreting various cytokines, while promoting the functions of Th17, Treg, TAMs cells, and can promote angiogenesis and metastasis.

MDSC is thought to be a cancer-promoting immune cell in the HCC tumor microenvironment and was discovered a decade ago (Gabrilovich et al., 2007). MDSC is divided into granulocyte or multinuclear MDSC(PMN MDSC) and mononuclear MDSC(M-MDSC) (Veglia et al., 2018). MDSCs enhance angiogenesis by producing vascular endothelial growth factor (VEGF), actuator 2 and MMP9. They can also cause cancer cells to migrate to endothelial cells and encourage metastasis (Zhou et al., 2018a). MDSC also suppresses T cell activity by secreting immunosuppressive cytokines, inducible nitric oxide synthase, and argininase (Gabrilovich et al., 2012; Gabrilovich, 2017). MDSCs can have a dual influence on immune cells *via* distinct methods. B cells, T cells, DCs and NK cells, are all inhibited by MDSCs. MDSCs, on the other hand, can stimulate Th17 cells, Tregs, and TAMs, as well as tumor angiogenesis and metastasis (Figure 3). There has been a great deal of research on the processes through which MDSC supports the advancement of HCC, and many therapeutic pathways have been developed for MDSC as a target of HCC. For example, myeloid-derived suppressor cells (MDSCs) are drawn to the tumor microenvironment by PIWIL1-overexpressed HCC cells. MDSCs consumption reduced the proliferation and growth of PIWIL1-overexpressed HCC tumors. Complement C3 stimulates HCC cell secretion *via* PIWIL1 and mediates the contact between HCC cells and MDSC *via* p38 MAPK activation in MD38, and then initiates the expression of immunosuppressive cytokine IL10. PIWIL1, which is expressed by tumor cells, could be a viable target for the development of new HCC treatments (Wang et al., 2021a). Tumor-infiltrating LY6G MDSCs from orthotopic liver tumors treated with sorafenib dramatically increased CD4 T cells expressing IL-10 and TGF-and decreased CD8 T cell cytotoxicity. *In vitro*, IL-6 protects LY6G MDSC from sorafenib-induced cell death. Combining sorafenib and anti-IL-6 antibody or anti-LY6G antibody dramatically decreased the cell proportion of LY6G MDSCs in orthotopic liver tumors, synergistically boosted sorafenib’s therapeutic efficacy and increased T cell proliferation (Chang et al., 2018). Icaritin blocks MDSC production by blocking the attenuation of EMH, thereby inhibiting the immunosuppressive effect of the tumor, thereby triggering an anti-tumor immune response. Therefore, Icaritin can be used as a new adjuvant or even as an independent therapeutic agent for the effective treatment of HCC(Tao et al., 2021). In Table 6, we show the relevant role of MDSC in HCC immune microenvironment in recent years.

**TABLE 6:** Tumor-promoting immune cells: Myeloid-derived suppressor cells.

Cells	The research direction	Result	References
MDSCs	The immune mechanism	The heterozygous loss of SPTBN1 significantly upregulated the liver expressions of IL-1α, IL-1β and IL-6, and increased the proportion of myeloid inhibitory cells (MDSC) and CD4CD25Foxp3 regulatory T cells (Foxp3Treg) in liver, which promoted the occurrence of HCC in DDC-fed mice.	Lin et al. (2021)
MDSCs	The immune mechanism	MDSCs contribute to tumor progression under psychological stress, chronic binding stress significantly promotes the growth of HCC, and MDSCs are mobilized from bone marrow to spleen and tumor sites. Also, the β-adrenergic signaling cascade plays a key role in the mobilization and recruitment of MDSC under chronic confinement stress.	Cao et al. (2021)
MDSCs	immunotherapy	Icaritin blocks MDSC production by blocking the attenuation of EMH, thereby inhibiting the immunosuppressive effect of the tumor, thereby triggering an anti-tumor immune response. Therefore, Icaritin can be used as a new adjuvant or even as an independent therapeutic agent for the effective treatment of HCC.	Tao et al. (2021)
MDSCs	immunotherapy	PIWIL1 overexpressed HCC cells attract myeloid suppressor cells (MDSCs) to the tumor microenvironment. MDSCs consumption reduced the proliferation and growth of HCC tumors overexpressed by PIWIL1. Complement C3 induces the secretion of HCC cells through PIWIL1, mediates the interaction between HCC cells and MDSC by activating the P38 MAPK signal in MD38, and then initiates the expression of the immunosuppressive cytokine IL10.	Wang et al. (2021a)
MDSCs	immunotherapy	After tivozanib treatment, the baseline number or frequency of FOXP3treg, MDSC, and exhausted T cells decreased significantly more. This may help identify patients who may benefit from c-kit/SCF antagonism and provide guidance for improving the efficacy of tivozanib in combination with immunotherapy.	Kalathil et al. (2020)
MDSCs	The immune mechanism	CD8^+^ T cells, MDSCs, and M2 macrophages were particularly increased in the tumorigenic liver of NCoA 5 ± male mice. NCoA5 deficiency promotes a unique hepatic tumorigenetic microenvironment through p21WAF1/CIP1 overexpression, which metformin reverses.	Williams et al. (2020)
MDSCs	immunotherapy/The immune mechanism	HSC promoted migration of MDSC through the SDF-1/CXCR4 axis. Pretreatment of MDSC with CXCR4 inhibitors or injection of SDF-1 knockout HSC inhibited migration of MDSC to the spleen and liver of tumor-bearing mice.	Xu et al. (2019)
MDSCs	Prognostic marker	Patients with an increased T-effector/Treg ratio before treatment showed significant improvement in OS. ERK + FLT-3+ Treg and MDSCs were significantly reduced after sorafenib treatment. An increase in baseline Flt-3+ p-ERK + MDSC was associated with a patient survival benefit. Conclusion High baseline CD4^+^ T effector/Treg ratio may be an important biomarker for prognosis in HCC.	Kalathil et al. (2019)
MDSCs	immunotherapy	CCR2 antagonists inhibited the infiltration of TAM and MDSC and delayed tumor growth in tumors of mice expressing A3b and A3b. Mechanically, upregulation of A3B in HCC inhibs the global abundance of H3K27me3 and reduces the presence of H3K27me3 on the chemokine Ccl2 promoter by interacting with the multicomb repressor complex 2(PRC2), thus recruiting large amounts of TAM and MDSC.	Wang et al. (2019c)
MDSCs	immunotherapy	Activated HSC induces mononuclear intrinsic p38 MAPK signaling, which triggers enhancer reprogramming for M-MDSC development and immunosuppression. Treatment with p38 MAPK inhibitors eliminated HSC-M-MDSC crosstalk to prevent HCC growth. Combined with I-BET762 suppressed patient-derived M-MDSC, combined with anti-PD-L1 therapy synergically enhanced TIL, resulting in tumor eradication and prolonged survival in a fibrotic HCC mouse model.	Liu et al. (2020b)
MDSCs	The immune mechanism	RIP3 knockdown results in an increase in MDSCs and a decrease in interferon Δ- positive (IFN-γ) clusters of tumor-infiltrating lymphocytes (CD8) differentiated into 8 positive (CD8) cells in HCC tissue, thus promoting immune escape and HCC growth in immunologically competent mice.	Li et al. (2019b)
MDSCs	The immune mechanism	By down-regulating the expression of IDO1, the HS donor induced T effector cells and inhibited MDSCs, and effectively restricted the tumor development of H22 HCC tumor-bearing mice.	Yang et al. (2019c)
MDSCs	The immune mechanism	March-derived suppressor cells (MDSCs) and tumor-associated macrophages (TAM) from the tumor microenvironment contribute to the suppression of the CD8 T cell response.	Liu et al. (2018a)
MDSCs	immunotherapy	Adoptive transfer of CIK to tumor-bearing mice induced an increase in inflammatory mediators (e.g., CX3CL1, IL-13) and tumor-infiltrating MDSC in the tumor microenvironment, and MDSCs effectively inhibited the cytotoxic activity of CIKs *in vitro*. In contrast, treatment with PDE5 inhibitors reversed MDSC inhibition by Arg1 and iNOS, while systemic treatment with PDE5 inhibitors prevented MDSC accumulation in the tumor microenvironment after CIK cell treatment and increased its anti-tumor efficacy.	Yu et al. (2019b)
MDSCs	The immune mechanism	RT/IL-12 significantly reduced the accumulation of tumor-infiltrating myeloid suppressor cells (MDSC) and its inhibitory function by reducing the production of reactive oxygen species	Wu et al. (2018)
MDSCs	immunotherapy	Tumor-infiltrating LY6G MDSCs from orthotopic liver tumors treated with sorafenib significantly induced CD4+T cells expressing IL-10 and TGF-β and down-regulated the cytotoxic activity of CD8 T cells. IL-6 protects LY6G MDSC against sorafenib induced cell death *in vitro*. The combination of anti-LY6G antibody or anti-IL-6 antibody and sorafenib significantly reduced the cell proportion of LY6G MDSCs in orthotopic liver tumors, enhanced the proliferation of T cells, and synergically improved the therapeutic effect of sorafenib.	Chang et al. (2018)
MDSCs	The immune mechanism	Tumor-infiltrating CD11BCD33HLA-DR MDSCs in HCC patients can effectively inhibit autologous CD8T cell proliferation. Concordant overexpression of CCRK and MDSC markers (CD11b/CD33) was positively associated with poorer survival. Hepatocyte CCRK stimulated the immunosuppressive CD11BCD33HLA-DR MDSC amplification of human peripheral blood mononuclear cells by up-regulating IL-6.	Zhou et al. (2018b)
MDSCs	The immune mechanism	In HCC, hypoxia induces the expression of ENTPD2 on cancer cells, leading to an increase in extracellular 5′-AMP, which in turn promotes MDSC maintenance by preventing its differentiation.	Chiu et al. (2017)
MDSCs	immunotherapy	The tumor suppressive effects of chemerin were associated with metastasis of tumor-infiltrating immune cells from myeloid suppressor cells (MDSC) to interferon-γ T cells and decreased tumor angiogenesis.	Lin et al. (2017)
MDSCs	The immune mechanism	TAF-derived cytokines (such as IL-6 and SDF-1A) can induce MDSC generation and activation, and then weaken the human anti-tumor immune response, thus creating favorable conditions for the development of HCC.	Deng et al. (2017)
MDSCs	immunotherapy	The frequency of MDSC before treatment is a prognostic factor for HAIC prevention of HCC. Patients with lower MDSC frequency also had significantly longer overall survival.	Mizukoshi et al. (2016)
MDSCs	immunotherapy	The frequency of MDSC increased significantly in HCC patients. It is associated with tumor progression, but not with liver fibrosis or inflammation.	Arihara et al. (2013)
MDSCs	The immune mechanism	The MDSC-mediated functional inhibition of NK cells mainly depends on NKP30 on NK cells.	Hoechst et al. (2009)

### Cancer-Associated fibroblasts

CAF is not an immune cell, but it plays an important role in the tumor microenvironment, so we will introduce it in detail here. CAF exists as a prominent component of the tumor stroma between various inflammatory cells and components in the tumor microenvironment (Kalluri, 2016). As a result, CAF possesses functions that normal fibroblasts do not. A vast number of prior studies have demonstrated that CAF plays a critical function in changing the tumor microenvironment and driving the development of a variety of cancers (Jiang et al., 2017a; Zhao et al., 2017a; Deng et al., 2017; Pistore et al., 2017). CAF is also significant in HCC. Many studies have revealed the great role of CAF in the pathogenesis, progression, prognosis, treatment and other aspects of HCC. CAF-derived cardioctonutrient-like cytokine 1 (CLCF1) has been found in studies to stimulate the release of TGF-β and CXCL6 in tumor cells, consequently increasing tumor stem cell development in HCC-TME (Song et al., 2021). Other research have found that CCN2, EMA, and FAP expression may be involved in the activation of CAF in HCC, resulting in aggressive behavior. The substantial association between EMA-expressing tumor cells and FAP-expressing CAF, as well as their topographical proximity, suggests that there may be interplay between tumor epithelial and stromal cells in the HCC tumor microenvironment (Kim et al., 2014). Table 7 summarizes the current role of CAF in HCC.

**TABLE 7:** Tumor-promoting immune cells: cancer-associated fibroblasts.

Cells	The research direction	Result	Reference
CAF	The immune mechanism	Sulf2 secreted by HCC cells induces THE differentiation of HSC into CAF through the TGFβ1/Smad3 signaling pathway.	Wang et al. (2021b)
CAF	immunotherapy	CNP inhibits the HCC promotion of CAF by inhibiting several HCC promoting cytokines secreted by CAF expressing GPR68. Further studies have shown that the combination of CNP and existing anticancer agents has some potential in the treatment of intractable HCC associated with the activation of CAF.	Yamanaka et al. (2021)
CAF	The immune mechanism	CAF-derived cytokines enhance the progression and metastasis of HCC by activating the circRNA-miRNA-mRNA axis in tumor cells.	Liu et al. (2021)
CAF	The immune mechanism	In HCC-TME, CAF-derived cardioctonutrient-like cytokine 1(CLCF1) increases the secretion of CXCL6 and TGF-β in tumor cells, thereby promoting tumor stem cell growth.	Song et al. (2021)
CAF	immunotherapy	Coptidine blocked the secretion of CAF exosome CIRCCCT3 and significantly inhibited tumor growth of HepG2 cells in immunodeficient mice.	Lv et al. (2020)
CAF	The immune mechanism	MiR-150-3p was significantly reduced in CAFS-derived exosomes and inhibited HCC migration and invasion. MiR-150-3p was transferred from miR-150-3p transfected CAF to HCC cells *via* exosomes and eliminated HCC migration and invasion.	Yugawa et al. (2021)
CAF	The immune mechanism	IL-6 and HGF are key EMT-stimulating cytokines secreted by H-CAF.	Jia et al. (2020)
CAF	The immune mechanism	The proportion of CAFs was positively correlated with the expression of CD73 in HCC cells. The c-Met and MEK-ERK1/2 pathways are activated by HGF from CAF, which upregulates CD73 expression in HCC cells.	Peng et al. (2020)
CAF	immunotherapy	Endothelial sialic acid protein expressed by CAF is the main regulator of macrophage recruitment and polarization, and inhibition of endothelial sialic acid protein inhibition is a potential therapeutic strategy for HCC.	Yang et al. (2020)
CAF	Prognostic marker	CAFs can produce plGFPLGF, which is associated with tumor angiogenesis markers and predicts poor prognosis in HCC patients.	Liu et al. (2020c)
CAF	immunotherapy	CAFs can promote the dryness and metastasis of HCC cells, and blocking autophagy can significantly reduce the enhanced dryness of CAFs, suggesting that targeting HCC autophagy may be an effective strategy for the treatment of HCC.	Zhao et al. (2019)
CAF	The immune mechanism	LXRα agonists limit TGFβ-dependent CAF differentiation and may limit the growth of primary HCC.	Morén et al. (2019)
CAF	immunotherapy	RVD1 inhibits the stem cell characterization of CAF-induced EMT and HCC cells by inhibiting the secretion of COMP.	Sun et al. (2019b)
CAF	The immune mechanism	Endogenous and exogenous BMP4 activates liver fibroblasts, acquires the ability to secrete cytokines, and enhances the aggressiveness of HCC cells.	Mano et al. (2019)
CAF	The immune mechanism	CAFs promote self-renewal, chemotherapy resistance, metastasis and tumorigenicity of CD24 HCC cells. CAFs secreted HGF and IL6 promote the dryness of CD24 HCC cells by phosphorylation of STAT3.	Li et al. (2019c)
CAF	The immune mechanism	HCC-CAFs regulate the survival, activation, and function of neutrophils in HCC through the IL6-STAT3-PDL1 signaling cascade.	Cheng et al. (2018)
CAF	The immune mechanism	IL-6 secreted by CAF promotes stem cell-like properties in HCC cells by enhancing STAT3/Notch signaling.	Xiong et al. (2018)
CAF	The immune mechanism	CAF-induced Notch3 expression is responsible for the activation of LSD1 in CSC, thereby promoting its self-renewal in HCC.	Liu et al. (2018b)
CAF	The immune mechanism	CAF-mediated tumor progression in HCC is associated with the deletion of anti-tumor miR-320a in CAF exosomes.	Zhang et al. (2017)
CAF	The immune mechanism	MiR-101 eliminated SDF1 signal transduction cells by inhibiting the expression of SDF1 in CAF and inhibiting the expression of VE-cadherin in tumors.	Yang et al. (2016)
CAF	The immune mechanism	CCL2, CCL5, CCL7 and CXCL16 secreted by CAF promote HCC metastasis through synergistic activation of HH and TGF-β pathways in HCC cells.	Liu et al. (2016)
CAF	immunotherapy	Targeting the CAF-derived, HGF-mediated c-Met/FRA1/Hey1 cascade may be a therapeutic strategy for the treatment of HCC.	Lau et al. (2016)
CAF	The immune mechanism	When stimulated, CAF shows the potential to differentiate into adipocytes, osteoblasts, and pancreatic cells. When co-cultured with human HCC cell lines, CAF upregulated the expression of TGFB1 and FAP genes in Huh-7 and JHH-6, thus having the ability to enter the circulation.	Sukowati et al. (2015)
CAF	The immune mechanism	The expression of CCN2, EMA and FAP may be involved in the activation of CAF in HCC, leading to aggressive behavior. The significant correlation between tumor cells expressing EMA and CAF expressing FAP and their terrain proximity suggests that there may be cross-talk between tumor epithelial cells and stromal cells in the HCC tumor microenvironment.	Kim et al. (2014)

### M2-Polarized Macrophages

**FIGURE 4:**
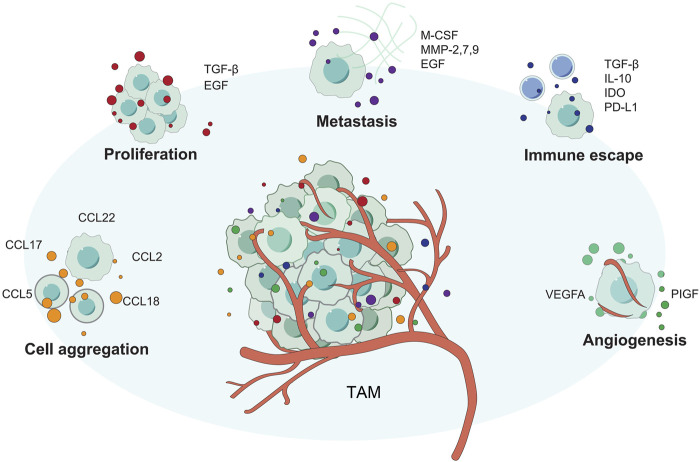
The different ways that TAM supports tumor growth. TAM promotes tumor proliferation, invasion, angiogenesis, and immune escape through various methods.

M2 polarized macrophages, as opposed to M1 polarized macrophages, have anti-inflammatory and pro-tumor actions (Figure 4). M2 macrophages are further differentiated into M2a, M2b, M2c, and M2d subsets. Th2 cytokines such as IL-13 and IL-4 can trigger macrophage transformation to the M2A phenotype, whereas TLR and immune complex activation induces M2B macrophages, the M2C subtype polarized by IL-10 (Hao et al., 2012; Sica and Mantovani, 2012). Despite the fact that there have been few research on M2-polarized macrophages in HCC, the function of them in the occurrence and development of HCC has been confirmed. According to several studies, arsenite raises miR-15b levels and causes M2 polarization in THP-1 cells. Increased miR-15b in Evs transfer from arsenite-treated THP-1 (AS-THP-1) cells to HCC cells *via* miR-15b. By targeting LATS1, it can reduce Hippo pathway activation while still accelerating the invasion and metastasis of growing HCC cells (Li et al., 2021). The potential therapeutic potential of M2 polarized macrophages has also been pointed out that Tumor cell-derived Wnt ligands induce M2-like polarization of TAM *via* traditional Wnt/-catenin signaling, resulting in tumor migration, development, immunosuppression and metastasis in HCC(Yang et al., 2018a). We summarize the current relevant research progress in Table 8.

**TABLE 8:** Tumor-promoting immune cells: M2-polarized macrophages.

Cells	The research direction	Result	Reference
M2-polarized macrophages	The immune mechanism	MicroRNA (miR-17-92) clusters from the extracellular vesicles (EVs) of M2-polarized tumor-associated macrophages (M2-TAMs) stimulate the imbalance of the TGF-β1/BMP-7 pathway, TGF-β1/BMP-7 pathway imbalance significantly promotes HCC cell invasion and stem cells by increasing the expression of differentiation inhibitor 1(ID1).	Ning et al. (2021)
M2-polarized macrophages	The immune mechanism	Elevated levels of Mir-15B are transferred from arsenite-treated THP-1 (AS-THP-1) cells to HCC cells *via* Mir-15b in EVs, inhibit Hippo pathway activation by targeting LATS1, and participate in the migration and invasion of proliferation-promoting HCC cells.	Li et al. (2021)
M2-polarized macrophages	The immune mechanism	β2-AR promotes the occurrence and development of HCC by silencing GRK2 in M2 polarized macrophages.	Wu et al. (2019b)
M2-polarized macrophages	immunotherapy	Tumor cell-derived Wnt ligands stimulate M2-like polarization of TAM through classical Wnt/β-catenin signaling, which leads to tumor growth, migration, metastasis, and immunosuppression in HCC.	Yang et al. (2018a)
M2-polarized macrophages	immunotherapy	M2-polarized macrophages promote the migration and EMT of HCC cells through the TLR4/STAT3 signaling pathway, suggesting that TLR4 may be a new therapeutic target.	Yao et al. (2018)

## The Controversial Immune Cell Type in Cancer: B Cells

One type of immune cells that cannot be ignored in the HCC tumor microenvironment is B cells. According to the current relevant studies, it is not clear whether B cells are “good” or “bad”. Some studies have reported that B cells promote HCC, while others have reported the opposite effect. B cells, on the one hand, release cytokines that comport with CTL activity and serve as potent antigen-presenting cells (APCs). On the other hand, they may be tumorigenic due to the production of cytokines that attract MDSC and promote angiogenesis (de Visser et al., 2005; Tsou et al., 2016). Studies have shown that CCL20 derived from tumor cells interacts with CD19CD5 B cells overexpressed by CCR6 to promote the development of HCC, possibly through enhanced angiogenesis (He et al., 2017). Liu et al. 's study confirmed that Selective recruitment of CXCR3 (+) B cells Bridges the pro-inflammatory interleukin-17 response and the polarization of tumorigenic macrophages in the tumor environment, and blocking the migration or function of CXCR3+ B cells may help to overcome HCC (Liu et al., 2015). Therefore, B cells are involved in both the development and inhibition of HCC. Table 9 highlights the most recent reliable research on the involvement of B cells in HCC.

**TABLE 9:** The controversial immune cell type in cancer: B cells.

Cells	The research direction	Result	Reference
B cells	Prognostic marker	CD20 B cells, naive B cells, and CD27 isotypic transformed memory B cells are independent prognostic factors for survival in HCC. During the progression of HCC, intratumoral infiltration of B cells is significantly impaired. High density of tumor-infiltrating B cells means better clinical outcome.	Zhang et al. (2019)
B cells	The immune mechanism	In mice with HCC, B cell depletion blocked the production of these macrophages, increased anti-tumor T cell response, and reduced HCC growth. This pathway is involved in the increased expression of DNA methyltransferase 1 and EZH2 in HCC and HCC cells.	Wei et al. (2019)
B cells	Prognostic marker	Tumor derived exosome-activated B cells strongly express Tim-1 protein and have inhibitory activity against CD8 T cells. Exogenous HMGB1 activates B cells and promotes the expansion of Tim-1 Breg cells through Toll-like receptor (TLR) 2/4 and mitogen-activated protein kinase (MAPK) signaling pathways. Accumulation of TIM-1BREG cells in tumors is associated with advanced disease, early recurrence, and reduced survival in HCC patients.	Ye et al. (2018)
B cells	The immune mechanism	In MDR2 mice, CD20 B cell ablation promoted age-mediated fibrosis regression and inhibited the tumorigenic TNFα/NF-κB pathway.	Faggioli et al. (2018)
B cells	The immune mechanism	B cells and IgG2 may play an important role in the inhibition of liver tumorigenesis. Hepatocellular specific expression of ras oncogene may play a role in the inhibition of B cells, and B cells and T cells may be inhibited in developing liver tumors.	Wang et al. (2017)
B cells	The immune mechanism	CCL20 derived from tumor cells interacts with CD19CD5 B cells overexpressed by CCR6 to promote the development of HCC, possibly through enhanced angiogenesis.	He et al. (2017)
B cells	Prognostic marker	More than 50% of B cells in HCC exhibit FcγRII-activated phenotypes, and the production of FcγRII-activated B cells may represent a mechanism through which immune activation is associated with immune tolerance in the tumor environment.	Ouyang et al. (2016)
B cells	The immune mechanism	A novel oncogenic PD-1(HI) B cell subtype has been identified in human HCC that exhibits a phenotype distinct from that of surrounding regulatory B cells. TLR4-mediated upregulation of BCL6 is critical for inducing PD-1(HI) B cells, which act through an IL10-dependent pathway after interacting with PD-L1, thereby causing T cell dysfunction and promoting disease progression.	Xiao et al. (2016)
B cells	Prognostic marker	Tumor infiltrating T cells and B cells are in close contact with each other, and their density is associated with superior survival in patients with HCC.	Garnelo et al. (2017)
B cells	The immune mechanism	Selective recruitment of CXCR3 (+) B cells Bridges the pro-inflammatory interleukin-17 response and the polarization of tumorigenic macrophages in the tumor environment, and blocking the migration or function of CXCR3 (+) B cells may contribute to the defeat of HCC.	Liu et al. (2015)
B cells	The immune mechanism	After liver MET transfection, TUNEL (+) hepatocytes were increased in B cell-or macrophage-deficient mice, suggesting that these cells provide a protective effect against MET-induced hepatocyte apoptosis.	Subleski et al. (2015)
B cells	The immune mechanism	An increase in intrahepatic B cells at the edge of the tumor was positively associated with tumor invasion characteristics and more tumor recurrence. Bregs directly interact with HCC cells through the CD40/CD154 signaling pathway, thereby promoting the growth and aggressiveness of HCC.	Shao et al. (2014)
B cells	The immune mechanism	As a trans activator, HBx can regulate the activated B nuclear factor kappa-light chain enhancer (NF-κB) and transcription factor AP-2. HBx may cause the loss of apoptotic function, or directly contribute to carcinogenesis by realizing the transformation function, and accelerate the development of HCC.	Zhang et al. (2014)
B cells	The immune mechanism	Activation-induced cytidine deaminase (AID) acts as genomic mutants in activated B cells, and inappropriate expression of AID is associated with immunopathological phenotypes of human B cell malignancies. Abnormal activation of AID in hepatocytes leads to the accumulation of multiple genetic changes in the p53 gene, which may enhance genetic susceptibility to mutagenesis leading to HCC development.	Kou et al. (2007)

## Conclusions and Perspectives

With the development of single-cell sequencing and other technologies, we have the opportunity to further explore TME. Immunotherapy, a new tumor treatment method, also has a better and broader application prospect. However, at present, it is still too early for immunotherapy to replace traditional chemotherapeutic therapy, and there is still a long way to go in the process of clinical application. However, immunotherapy can be regarded as a good alternative therapy for patients with chemotherapy resistance. As mentioned in this paper, immunotherapies for tumor suppressor related immune cells, such as effect DCs, as well as tumor promoting immune cells, such as MDSC/Treg, are being developed one after another.

Compared with traditional chemotherapy, immunotherapy has many advantages. For example, immunotherapy has fewer overall side effects than chemotherapy and, once effective, may lead to long-term survival and even clinical cure. In addition, immunotherapy can be used as an important anti-tumor adjuvant therapy in addition to chemotherapy, radiotherapy and surgery. Appropriate immunotherapy can kill the tiny residual tumor cells after chemotherapy or some tumor cells resistant to chemotherapy. Immunotherapy should be considered for patients who are intolerant to chemotherapy or have extensive metastasis and cannot undergo surgery, radiotherapy or chemotherapy (Yang, 2015).

At present, the mainstream immunotherapy mainly includes CAR T therapy/immune checkpoint inhibitor therapy (PD-1, PD-L1, etc.)/tumor vaccine. CAR-T is the T cells, biological engineering, when the cancer has an immune deficiency, immune surveillance, give play to the role of the case, through the biological engineering to determine the targets of leukemia, it specifically chimeric in T cells, to attack the leukemia cells, the effect is significant, but easy to appear “storm” cells, serious and even cause death (Feins et al., 2019). Immune checkpoint inhibitor therapy has the advantage of long-term survival and relatively small adverse reactions. This therapy activates tumor-specific immune cells in the body by removing or attenuating the negative regulatory factors of immunoreactive cells, but it is not suitable for all patients. The higher the mutation load, the better the treatment response. Therefore, biomarkers should be used to screen the dominant population. The most common predictive indicators of PD-1/PD-L1 immune checkpoint inhibitors are microsatellite instability, PD-L1 and tumor mutation load (Pinato et al., 2019). The treatment of cancer vaccines is still incomplete.

Immunotherapy is not the end of tumor therapy; on the contrary, tumor immunotherapy represented by immune checkpoints has just opened a new chapter in tumor therapy. Combined with the basic and characteristics of immunotherapy, with the deepening of human understanding of tumors, tumors as a chronic disease that can be cured are no longer so far out of reach.
